# HerpesDRG: a comprehensive resource for human herpesvirus antiviral drug resistance genotyping

**DOI:** 10.1186/s12859-024-05885-5

**Published:** 2024-08-27

**Authors:** O. J. Charles, C. Venturini, R. A. Goldstein, J. Breuer

**Affiliations:** 1 Department of Infection, Immunity and Inflammation, University College London, Great Ormond Street Institute of Child Health, London, WC1N 1EH UK; 2 Division of Infection and Immunity, University College London, London, WC1E 6BT UK; 3 Great Ormond Street Hospital for Children National Health Service Foundation Trust, London, WC1N 1LE UK

**Keywords:** Herpes, Drug-resistance, Database, CMV, VZV, Ganciclovir, Letermovir, Aciclovir, Amenamevir

## Abstract

The prevention and treatment of many herpesvirus associated diseases is based on the utilization of antiviral therapies, however therapeutic success is limited by the development of drug resistance. Currently no single database cataloguing resistance mutations exists, which hampers the use of sequence data for patient management. We therefore developed HerpesDRG, a drug resistance mutation database that incorporates all the known resistance genes and current treatment options, built from a systematic review of available genotype to phenotype literature. The database is released along with an R package that provides a simple approach to resistance variant annotation and clinical implication analysis from common sanger and next generation sequencing data. This represents the first openly available and community maintainable database of drug resistance mutations for the human herpesviruses (HHV), developed for the community of researchers and clinicians tackling HHV drug resistance.

## Background

Human herpesviruses (HHV) and their associated diseases are a major health problem worldwide for immunocompromised patients. The prevention and treatment of human cytomegalovirus (CMV) and herpes simplex virus (HSV) associated diseases for example are essential in management of solid organ and hematopoietic stem cell transplant recipients [1–4]. Although a handful of treatment options including aciclovir, penciclovir (HSV) and ganciclovir, maribavir, letermovir (CMV) are available, resistance causing mutations are now found for all approved drugs and pose a significant threat due to aggressive disease course and a greater mortality risk [5–8].

Drug resistance in a clinical setting is often diagnosed by sequencing followed by characterisation of variants (genotyping), rather than isolating and phenotypically characterising (phenotyping) due to cost and time benefits [9]. Genotyping does however require that any variants present have previously been phenotyped. In these experiments a single novel mutation is transferred into a reference virus, or a clinical isolate is found with similar characteristics. The impact of this mutation on drug sensitivity can then be determined by a plaque reduction assay (PRA), Bacterial Artificial Chromosome reporter assay or similar, returning an IC_50_ against a given drug. It is typical to report the mutations impact on antiviral sensitivity as a fold change relative to the control strain IC_50_, where CMV is currently the only HHV with consensus guidelines relating fold change to a “resistant” or “susceptible” phenotype [10–13].

An issue is that those previously phenotyped data are published in a disparate collection of reports. This has meant that researchers often must perform a review of the scientific literature for any present mutations in a viral isolate, which is time consuming, especially when there are several reports for a given mutation [14, 15]. Therefore there have been published efforts to collate this data previously such as in reviews, however the data contained is not updateable and requires reformatting for use in informatics [9, 16]. Developing a comprehensive mutation phenotype map that is community maintainable and in an open format could play an important role in developing improved antiviral therapies [17, 18].

User-friendly tools are also important to enable researchers to make use of such databases [19–21]. Any such tool built to analyse HHV sequence data should support both Sanger consensus sequence outputs, as this is still widely adopted, as well as modern Next Generation Sequencing (NGS) variant outputs [22]. NGS has become a popular option and provides advantages such sensitivity to low-level variants that helps in early decision making [23–25] and evidence that accumulation of low-level variants may be associated with poor clinical outcome [26, 27]. Additionally there is interest in sequencing more widely than just the DNA polymerases (HSV1/2:UL30, CMV:UL54, varicella-zoster virus (VZV):ORF28) and serine/threonine kinases (HSV1/2: UL30, CMV:UL97, VZV:ORF36) genes as the number of antiviral targets has grown [9, 28, 29]. For example CMV mutants in UL51, UL56 and UL89 which are associated with letermovir resistance are not sequenced in many diagnostic labs [10].

Here we present an open-source comprehensive database that links HHV mutations to impact on drug sensitivity. This resource, which we have called Herpes Drug Resistance Genotyping “HerpesDRG”, is interrogatable and maintainable by the community as the database and tools are released under permissive MIT licenses on GitHub, enabling anyone to discuss and propose alterations. The database is released along with analytical tools suitable for resistance annotation of both sanger and NGS sequence data from CMV, HSV1, HSV2, VZV and HHV6. To the best of our knowledge this database differs from comparable resources as the latter do not include all genes targeted by current clinically relevant drugs, are unable to update data after publication or are closed-source and thus inaccessible to community verification [9, 16, 19].

HerpesDRG currently contains 1341 unique mutations across 17 genes, including 6 HCMV genes and 4 HSV-1 genes. No comparable resource contains HCMV genes UL27, UL51 or HSV-1 genes UL5, UL52, nor do they consider the latest antiviral treatments such as pritelivir and maribavir. Even for the genes covered in existing databases, HerpesDRG is the most comprehensive (i.e. 289 unique HSV-1 UL23 mutations versus 243 in [16]).

## Construction and content

### Database construction

Literature regarding mutation resistance phenotype were identified by a comprehensive PubMed [30] search with key terms “Cytomegalovirus [Title] AND resistance [Title/Abstract]”, likewise for the other HHVs. Papers were inspected with regular expressions to detect the occurrence of mutations, from where literature article data was manually extracted. Each entry in the database represents the relationship between a mutation, study, control species, assay method, metadata, and fold change, where fold change values are stored for 11 currently clinically important drugs. Multiple entries may be present for the same variant where either a single publication provides different test methodology to produce independent sensitivity results, or where the same variant is tested across multiple publications. Fold change values come in numeric form if fold changes are possible to extract, or as simply “Resistance” or “Polymorphism” if only a phenotype is described. Study reference data comes in the form of a title, a HTTP link and a DOI. Assay information columns record the method of strain generation, i.e., “marker transfer”, or “isolated strain”, the test method such as “PRA” or “dye uptake assay” and the control strain used to generate a fold change. For some well-studied mutations, co-occurring mutations have been observed to amplify resistance and are recorded where relevant [31, 32]. Finally, any metadata such as when the entry was created, any notes and a status flag. Only rows with the status “A” for active are returned in the R package and webserver.

It is well understood that a single key mutation is almost always the cause of drug resistance for the HHV’s, while other mutations present in phenotypically resistant strains are neutral polymorphisms [9, 15, 33]. In addition to phenotyped viruses with introduced single mutations, this database also contains entries from clinical isolates with multiple co-occurring novel mutations. We use the following rules when including these entries: (a) If the strain is sensitive in vitro, any uncharacterised mutations are accepted as neutral polymorphisms. (b) Where a strain has a single uncharacterised mutation, and the others are known neutral polymorphisms, the single unknown mutation is entered with the strain’s fold change. (c) If there are multiple uncharacterised mutations the entry is recorded along with co-mutations and set the status “O” for obsolete, it will not feature in processes. (d) If at a later date, with new data, only a single uncharacterised mutation now is present for an entry as in (c), the entry is re-looked at and treated as in (b).

As of the date of publication, the database contains 1786 unique entries of which 1341 are unique mutations. Drug sensitivities are recorded for ganciclovir, aciclovir, cidofovir, foscarnet, brincidofovir, letermovir, brivudine, penciclovir, tomeglovir, maribavir, cyclopropavir, amenamevir and pritelivir.

## Utility and discussion

### Drug resistance genotyping with HerpesDRG

**Figure 1:**
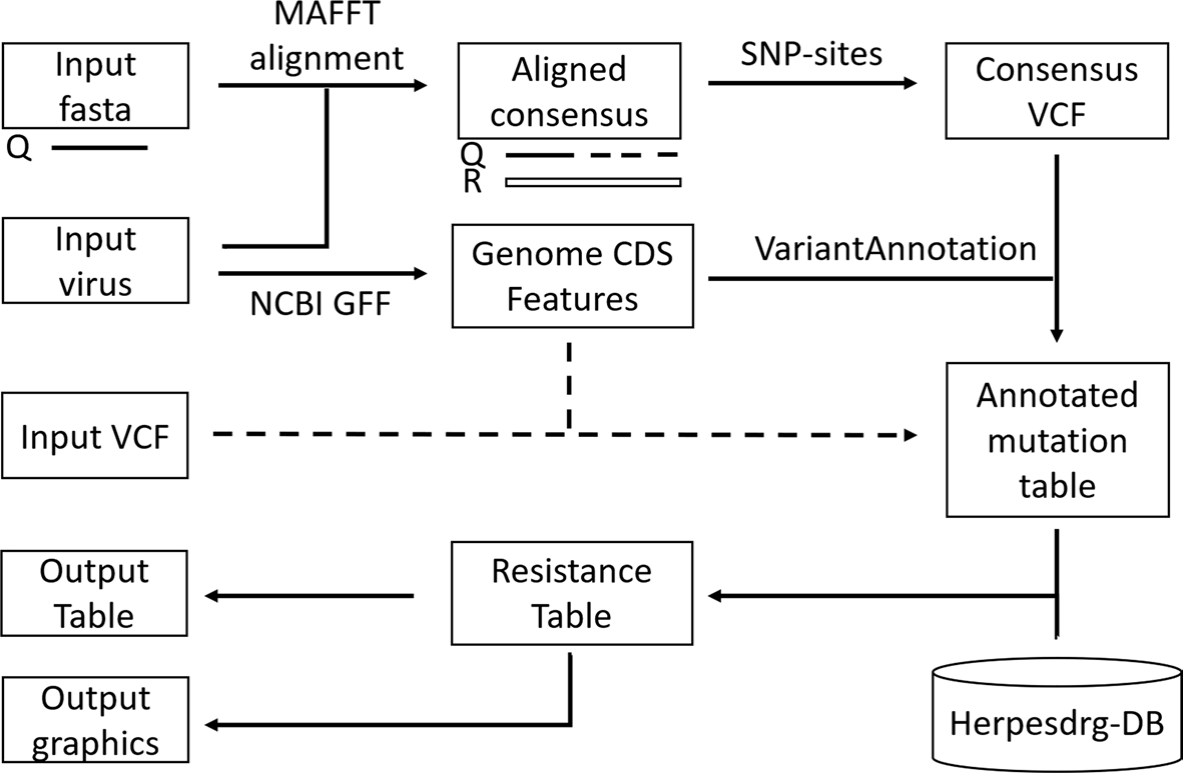
Graphical representation of the HerpesDRG annotation of FASTA sequence input or VCF inputs (dashed lines). *Q* Query sequence, *R* NCBI Reference sequence

To facilitate the resistance annotation of virus sequence data we developed HerpesDRG, a R package that enables the assessment of resistance mutations from standard input files available from 1st, 2nd and 3rd generation sequencing technologies (i.e. VCF or FASTA files) (Fig. 1). FASTA format sequences are aligned to the selected viruses reference genome with MAFFT [34] using the “—add—keeplength” parameters, such that both whole genome sequences or genetic fragments (e.g. UL23, UL54) can be inputted. SNP-sites is used to extract SNP’s and downstream functions identify indels [35]. For detection of low frequency mutations Variant Call Format (VCF >  = version4.0) and default Varscan2 output file are accepted, where the data has been mapped to the relevant NCBI reference strain [36]. Quality control should be applied as necessary prior to the use of HerpesDRG. All input formats are converted to a variant which is processed using the VariantAnnotation R package to predict their effect on coding regions [37, 38]. The HerpesDRG “call_resistance” function produces the key output, an annotated variant table including resistance data present in the database. In the case of a mutation present having multiple entries in the database, all relevant entries are returned.

**Figure 2:**
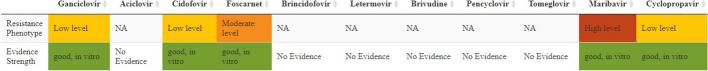
Clinical overview table produced by the “make_clin_table” function for the example data A10.vcf. Example code on GitHub

A concise clinical output can then be generated from this table using the “make_clin_table” function (Fig. 2). Here for each drug HerpesDRG identifies the mutation of maximal fold change found in the sample present at greater than 10% frequency, then allocates a category to that drug accordingly: High level (maximum fold change above 15), Moderate level (maximal fold change between 5 and 15), Low level (the maximum fold change between 2 and 5), Polymorphism (less than 2, or recorded as such), Resistant (only anecdotal resistant data was returned), NA (no mutations returned) and evidence strength as: “Good, in vitro”, or NA (no evidence). These fold change cut-off values are in line with recommendations from ‘The Third International Consensus Guidelines on the Management of Cytomegalovirus in Solid-organ Transplantation’ [10] which we use consistently across all viruses.

### Usage

Install HerpesDRG by running the R command: devtools::install_github("ojcharles/herpesdrg”). This will take care of installing other dependencies and the database. Inputs for the “call_resistance” function takes as input any of the aforementioned file types, along with which virus to genotype against and flags for whether to return all synonymous/non-synonymous mutations (default is only resistance mutants). The above examples were all based on CMV for consistency but work equivalently for the other herpesviruses.

The HerpesDRG toolset is also accessible through a user-friendly web interface included in the R package and available at cmv-resistance.ucl.ac.uk/herpesdrg/. Here users upload sequence data as described above and select the virus of interest from the drop-down list. There the full resistance mutations table, the simplified clinical overview and additional graphics are generated automatically. Specific terms of use for the hosted instance are included with the shiny application. The application was developed using the shiny framework [39]. We include example files for whole genome analysis (VCF, varscan tab, FASTA), and specific gene regions FASTA.

## Conclusions

We have developed a comprehensive, open-source database detailing drug resistance mutations observed in the antiviral treated human herpesviruses. This database which can be maintained collaboratively by the community, is paired with an intuitive tool making the analysis of resistance in sequencing data more accessible for clinical users; such as in Great Ormond Street Hospital [27, 40, 41].

## Data Availability

The HerpesDRG database generated during the current study is available under a MIT license at the GitHub repository ojcharles/herpesdrg-db. The R package for resistance genotyping is available under a MIT license at the GitHub repository ojcharles/herpesdrg. A user-friendly webserver for resistance genotyping is available at cmv-resistance.ucl.ac.uk/herpesdrg.

## Abbreviations

HHV: Human herpesviruses
CMV: Human cytomegalovirus
HSV: Herpes simplex virus
PRA: Plaque reduction assay
VZV: Varicella-zoster virus
ORF: Open reading frame
NGS: Next Generation Sequencing
